# Gabapentinoid detection in coronial casework in Gold Coast, Australia: a 5-year retrospective study

**DOI:** 10.1007/s12024-023-00694-3

**Published:** 2023-08-14

**Authors:** Isabella Thompson, Zeena Gadsby, Jeremy Martin, Melissa Thompson, Rexson Tse

**Affiliations:** 1 Gold Coast University Hospital, Southport, QLD Australia; 2 Griffith University School of Medicine, Southport, QLD Australia; 3 Forensic and Scientific Services, Health Support Queensland, Gold Coast University Hospital, 1 Hospital Boulevard, Southport, QLD Australia

**Keywords:** Pathology, Postmortem, Toxicology, Drug death, Gabapentinoids, Pregabalin

## Abstract

Gabapentinoids is a class of drug with analgesic, anxiolytic, and anticonvulsant properties and has a reported increase in prescription, use, and adverse outcomes. Regional studies are scant, and postmortem toxicological data may characterise patterns of regional use and inform local interventions. Characterising drug and non-drug-related deaths with gabapentinoid detection may also aid in toxicology interpretation. A 5-year retrospective study on all deaths admitted to the Gold Coast University Hospital under where toxicological analysis was performed. Of the gabapentinoids, only pregabalin was detected over the study period, and annual rates of detection did not differ significantly over the period (7.4–12.4%). In cases where pregabalin was detected, it was 15 times more likely to be a drug-related death. Drug-related deaths where pregabalin was detected have higher levels of pregabalin, are younger, and had a greater proportion of concurrent opioid detection. Postmortem detection of pregabalin was associated with drug-related deaths. Higher levels, younger decedents, and concurrent use of opioids were found in drug-related deaths. Public health interventions and regulated prescribing to target concurrent pregabalin and opioid use may address the burden of pregabalin drug-related deaths.

## Introduction

Gabapentinoids are a class of drugs that bear structural similarities to *gamma*-aminobutyric acid and have analgesic, anxiolytic, and anticonvulsant properties. Thus, they are used to treat epilepsy, anxiety disorder, and neuropathic pain [1]. These drugs are prescription-only-medication in Australia with reported low toxicity [1, 2]. Under the Pharmaceutical Benefits Scheme (PBS), only two gabapentinoids are available in Australia being gabapentin and pregabalin (https://www.pbs.gov.au/browse/body-system?depth=4&codes=n02bf#n02bf). Gabapentinoids have the potential for misuse due to the effects of euphoria and relaxation, described as simulating drunkenness [3]. These deleterious effects are potentiated when taken in combination with other agents exhibiting similar effects, such as with opioids [4–7].

Nationally, Australia has recorded a substantial increase in prescription, use, and adverse outcomes since this drug class first arrived on the Australian market in 2005 [2, 8–10]. Most postmortem studies on gabapentinoids focused on fatality with scant literature reporting on overall trends and detection in postmortem investigation [11–14]. Recent studies have documented divergence in local and national trends in drug-related deaths [15]. Thus, examining local gabapentinoid detection may aid in understanding the background use of these drugs. Furthermore, due to the increased prescription of gabapentinoids, its level can be difficult to interpret. Comparing characteristics of drug and non-drug-related deaths when gabapentinoids are detected may aid in its interpretation.

This 5-year retrospective study was carried out in Gold Coast, Australia, to investigate postmortem gabapentinoid detection, specifically to:

1. Document the prevalence and trend of gabapentinoid detection
2. Compare the characteristics of drug and non-drug deaths when gabapentinoids were detected

## Material and methods

### Case selection

A 5-year retrospective study (1 January 2017- 31 December 2021) at Gold Coast, Queensland, Australia, on all cases where toxicological analysis was performed. Toxicological analysis was performed in all unnatural deaths initially determined by the reporting police, and cases requested by the coroner/family/police, or the cause of death was uncertain at the postmortem examination.

Cases were identified using a manual search of our local database for all toxicological reports in the timeframe. For each case, sex, and age (years), whether it was a drug-related death, and whether gabapentinoids were detected, the specific gabapentinoid and its level were recorded. A drug-related death was defined as a death from acute (or acute on chronic) alcohol toxicity, and prescribed, illicit, and/or over the counter medications with or without alcohol being contributory. Toxic chemical-related death (e.g. carbon monoxide, hydrogen sulphide, sodium nitrite, ethylene glycol) was categorised as non-drug-related death. All samples for toxicological analysis were sent to an accredited toxicology laboratory. The analytical method for pregabalin (the only gabapentinoids detected in this study) was protein precipitation and LC/MS/MS and was validated for blood with the limit of detection of 0.05 mg/L and limit of quantification of 0.16 mg/L [16].

Other drugs detected were grouped into broad categories including opioids (prescribed and illicit), stimulants, benzodiazepine and zopiclone/Z-drugs, anti-depressants, antipsychotics, and alcohol.

Exclusion criteria are as follows:

1. All suspicious and homicide deaths due to potential legal implications
2. Paediatric (age < 18 years) and maternal deaths due to altered metabolism and confidentiality issues (low numbers)
3. Bodies that show advanced decomposition (cases with decomposition beyond local green discolouration of the abdomen were excluded).

### Statistical analysis

Statistical analysis was performed by an open-source statistical program via R studio, and a *p*-value of < 0.05 was considered significant. Continuous variables were presented as mean, median, standard deviation (s.d), minimum (min), and maximum (max). Categorical variables were presented as counts.

Spearman’s correlation test was used to examine the trend of gabapentinoid detection in both absolute numbers and in proportion of toxicological tests performed.

Chi-square tests were used to explore any differences in categorical variables. Student *t*-test or Mann–Whitney *U*-test was used to explore any differences in continuous variables depending on the distribution (Kolmogorov–Smirnov test). The relative risk of being a drug death when gabapentinoids were detected was calculated.

### Ethics approval

This study was approved by the Forensic Scientific Services Human Ethics Committee (FSS-HEC22-30).

## Results

During the 5-year period, a total of approximately 2300 cases were admitted to the Department of Forensic Pathology, Gold Coast, Queensland, Australia, and 916 cases were suitable for this study. In the 916 cases, the mean age was 48.1 years (median: 47, s.d: 18.0, min: 18, max: 94) with a male predominance (M:F = 637:279). Three hundred thirty-four (36%) cases were determined to be drug-related deaths.

### Overall trend in gabapentinoid detection

**Fig. 1:**
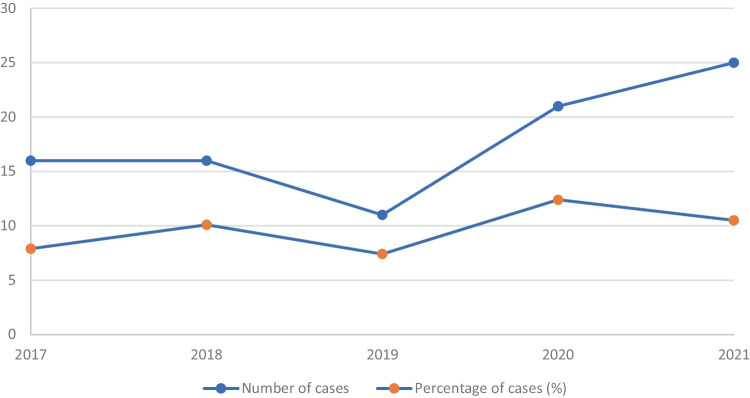
Prevalence of pregabalin present in toxicological testing in coronial case work in Gold Coast, Australia, between 2017 and 2021 (numbers of cases—absolute number of cases where pregabalin was detected; percentage of cases—percentage of cases where pregabalin was detected in all cases where toxicological analysis was performed)

Of the 916 cases, 89 (9.7%) contained gabapentinoids, exclusively pregabalin. From 2017 to 2021, the number of cases with gabapentinoids (pregabalin) per year detected was 16 (7.9%), 16 (10.1%), 11 (7.4%), 21 (12.4%), and 25 (10.5%), respectively (Fig. 1). Spearman’s correlation for absolute number and proportion of detected was 0.67 (*p* = 0.21) and 0.6 (*p* = 0.28), respectively.

### Characteristics of cases with gabapentinoids (pregabalin) detected

Of the 89 cases where pregabalin were detected, 76 cases were determined to be drug-related deaths, and 13 were non-drug-related deaths. The overall mean level of pregabalin for both drug-related and non-drug-related deaths was 10.0 mg/L (median: 6.5, s.d: 10.8, min: 0.2, max: 45). The cases of pregabalin detected in drug-related death (76 out of 258 cases) were significantly higher than that in non-drug-related death (13 out of 582) (chi square, *p* < 0.05). When pregabalin was detected, the relative risk in being a drug-related death was 15 times higher than being a non-drug-related death.

In the 76 cases of drug-related deaths, the mean age was 47.1 years (median: 46.5, s.d: 13.9, min: 18, max: 78) with a male predominance (M:F = 46:30). The mean level of pregabalin was 11.2 mg/L (median: 7, s.d: 11.2, min: 0.3, max: 45). There was concurrent detection of stimulants in 15 cases, opioids in 67 cases, benzodiazepines in 57 cases, zopiclone in 6 cases, antidepressants in 36 cases, antipsychotics in 11 cases, and alcohol (unrelated to postmortem artifact) in 22 cases.

In the 13 cases of non-drug-related deaths, the mean age was 57.5 years (median: 64, s.d: 20.0, min: 22, max: 80) with a slight male predominance (M:F = 7:6). The mean level of pregabalin was 2.8 mg/L (median: 1.9, s.d: 2.5, min: 0.2, max: 8). There was concurrent detection of stimulants in 2 cases, opioids in 1 case, benzodiazepines in 7 cases, zopiclone in no cases, antidepressants in 7 cases, antipsychotics in 2 cases, and alcohol (unrelated to postmortem artifact) in 1 case. The causes of death in these cases included cardiovascular causes, metabolic derangements (acute diabetic complications), trauma, and asphyxia (i.e. hanging, plastic asphyxia, choking on food).

Comparing drug and non-drug-related deaths when pregabalin was detected, decedents in drug-related deaths were significantly younger (*t*-test, *p* < 0.05), and pregabalin levels were significantly higher (Mann–Whitney U-test, *p* < 0.01), with higher presence of opioids (chi-square, *p* < 0.05). There was no significant difference in sex distribution, presence of stimulants, benzodiazepine, and zopiclone/Z-drugs, antidepressants, antipsychotics, and alcohol (chi-square, *p* > 0.05).

## Discussion

### Overall trend in gabapentinoids detection

This presented study showed that, in Gold Coast Australia, 9.7% of coronial cases where toxicological analysis was performed detected gabapentinoids, which was exclusively pregabalin. There were no significant changes in both case numbers and percentage detected in the study period which ranged between 16 and 25 cases per annum (7.4–12.4%).

Globally, there has been an increase in prescription of gabapentinoids despite rising harm from recreational use, poisoning, and death [8]. Canada observed a 22-fold increase in pregabalin prescribing from 2013 to 2014 [6], while both the USA and UK observed a two-fold increase between 2012 and 2016 [17] and 2013 and 2018 [18], respectively. A cross-sectional study of Australian general practice clinical data pre and post subsidisation of pregabalin in March, 2013 found an eightfold increase in the rate of pregabalin prescribing from 13 per 10,000 prescriptions (March, 2012–February, 2013) to 104 per 10,000 prescriptions by the equivalent period 5 years post subsidisation [8].

Despite the national and international increase in prescription rate, gabapentinoid/pregabalin detection in our region showed a steady trend. This may be due to different study period, regional variation, and different population groups (clinical vs. postmortem), and not all cases admitted had toxicological analysis. The trend reported in this study provides a ‘baseline’ detection in our region, which would allow future monitoring.

In terms of type of gabapentinoids detected, although both gabapentin and pregabalin are available in Australia, our study exclusively detected pregabalin. There are multiple reasons for this and may include differences in the relative toxicity of these drugs and/or prescribing characteristics. The literature suggests that pregabalin is more addictive over gabapentin, and current prescribing of pregabalin is fivefold that of gabapentin [13, 19]. Furthermore, in Australia, gabapentin is subsidised for epilepsy only, but not for neuropathic pain, whereas pregabalin is subsided for both conditions [13].

### Characteristics of cases with gabapentinoids detected

Detection of pregabalin was 15 times more likely in drug-related deaths. When pregabalin was detected, decedents were significantly younger, had higher levels of pregabalin, and had a greater proportion of opioids in drug-related deaths.

In Australia, pregabalin is subsidised by the Pharmaceutical Benefits Scheme (PBS) for the treatment of neuropathic pain refractory to treatment by other medicines (https://www.pbs.gov.au/medicine/item/2335X-2348N-2355Y-2363J). Despite this, Australian prescribing data between 2012 and 2018 suggests that more than half (50.3%) of patients lacked a diagnosis of neuropathic pain [8]. Concurrently, there was also an increase in same-day prescribing of central nervous system (CNS) depressants in Australia, whereby pregabalin was prescribed with an opioid to 24,554 patients (38.1%), benzodiazepine to 8435 patients (13.1%), and both medication classes to 4.4% of patients [8]. This shows that gabapentinoid/pregabalin has a clear therapeutic role but is at the risk of being abused in combination with other prescription medications. The interpretation of pregabalin can be challenging because the nature of it being commonly used in combination with other prescription medication. Comparing profiles between drug and non-drug-related deaths may enable/assist in the interpretation of pregabalin level in postmortem population and also identify potential characteristics to aid in the prevention of gabapentinoid abuse and drug-related deaths.

In the postmortem setting, there is no national data on the detection of gabapentinoids in non-drug-related deaths. Our regional data was able to report the basic variation in characteristics and gabapentinoid/pregabalin levels for both drug and non-drug-related death for comparison.

The epidemiology of drug-related deaths involving gabapentinoids at a regional level was similar to findings at a national level in terms of age and sex distribution. A recent retrospective case series leveraging data from the National Coronial Information System database between 2000 and 2020 reported a mean age of 45.7 years and a slight male predominance, with 55.2% of cases being male decedents [13]. This was comparable to our results.

The average blood concentration of pregabalin in drug-related deaths was also similar to national and international data, with a mean of 16.3 mg/L reported between 2000 and 2020 in Australia [13]. Data from a retrospective study of Finland deaths attributed to pregabalin abuse from 2010 to 2011 showed a median blood concentration of 15 mg/L [12]. While data from a UK study between 2012 and 2014 reported a lower median concentration of 8 mg/L [11]. Different from many drugs in the postmortem setting, pregabalin does not appear to exhibit significant postmortem redistribution [20]. The pregabalin level in drug-related death was significantly higher than non-drug-related deaths but had an overlap in the lower levels. This suggests that pregabalin levels in drug-related death can be at therapeutic levels. Thus, interpreting its level alone might have limited utility in ascribing a drug-related death.

Consistent with national and international trends, gabapentinoids were rarely detected in isolation in postmortem cases, with an overwhelming predominance of other CNS depressants such as opioids, benzodiazepines, antidepressants, and antipsychotic drugs. Australian postmortem toxicological analysis from 2000 to 2020 found 90.1% of cases with concomitant detection of opioids, 76.9% hypnosedatives, and antidepressants 60.5% [13]. Similarly, Finland postmortem data from 2010 to 2011 found that 91% of decedents had used opioids simultaneously while USA postmortem data from 2019 to 2020 observed 85–90% of gabapentinoid death-involved opioids [21]. This is unsurprising given that the co-prescribing of other CNS depressants increases the risk of toxicity of gabapentinoids [8, 13].

In our study, apart from pregabalin detection, there was significant epidemiological and drug profile differences between the two groups. The drug-related death group was younger and had a higher proportion of opioid detection. These differences, together with pregabalin level, may assist in determining when a death was drug-related or not. Also, this difference may provide insight in preventing gabapentinoid abuse and drug-related deaths by targeting a younger population and managing the risk of abuse in concurrent prescription of CNS depressant such as opioids.

## Conclusion

This 5-year retrospective study showed that, in Gold Coast, Australia, gabapentinoid detection in postmortem coronial casework was exclusively pregabalin with no significant changes in both case numbers and percentage detected. In postmortem cases where pregabalin was detected, they were 15 times more likely to be a drug-related death. Drug-related deaths where pregabalin was detected had higher levels of pregabalin, were younger decedents, and had a greater proportion of concurrent opioid detection compared to non-drug-related deaths.

## Key points

1. Pregabalin was the only gabapentinoid detected in this study.
2. No significant changes in pregabalin detection in the 5-year study period.
3. Pregabalin detection was 15 times more likely to be a drug-related death.
4. Drug-related deaths with pregabalin detected had higher levels, are younger, and had higher opioid detection.

## Data Availability

Data can be made aviailable through requesting the corresponding author.
